# Longitudinal neuroanatomical and behavioral analyses show phenotypic drift and variability in the Ts65Dn mouse model of Down syndrome

**DOI:** 10.1242/dmm.046243

**Published:** 2020-09-25

**Authors:** Patricia R. Shaw, Jenny A. Klein, Nadine M. Aziz, Tarik F. Haydar

**Affiliations:** 1 Graduate Program in Neuroscience, Boston University School of Medicine, Boston, MA 02118, USA; 2 Department of Anatomy and Neurobiology, Boston University School of Medicine, Boston, MA 02118, USA; 3 Center for Neuroscience Research, Children's National Hospital, Washington, DC 20010, USA

**Keywords:** Developmental disorders, Brain development, Milestones, Adult behavior, Oligodendrocytes

## Abstract

Mouse models of Down syndrome (DS) have been invaluable tools for advancing knowledge of the underlying mechanisms of intellectual disability in people with DS. The Ts(17^16^)65Dn (Ts65Dn) mouse is one of the most commonly used models as it recapitulates many of the phenotypes seen in individuals with DS, including neuroanatomical changes and impaired learning and memory. In this study, we use rigorous metrics to evaluate multiple cohorts of Ts65Dn ranging from 2014 to the present, including a stock of animals recovered from embryos frozen within ten generations after the colony was first created in 2010. Through quantification of prenatal and postnatal brain development and several behavioral tasks, our results provide a comprehensive comparison of Ts65Dn across time and show a significant amount of variability both across cohorts as well as within cohorts. The inconsistent phenotypes in Ts65Dn mice highlight specific cautions and caveats for use of this model. We outline important steps for ensuring responsible use of Ts65Dn in future research.

This article has an associated First Person interview with the first author of the paper.

## Abstract (precis)

**Summary:** Comparative analyses of temporal cohorts of the Ts65Dn mouse model of Down syndrome reveal phenotypic variability affecting neurodevelopment and learning and memory behaviors, calling into question the validity of this model.

## INTRODUCTION

Down syndrome (DS) is caused by the triplication of chromosome 21 (HSA21) (Lejeune et al., 1959) and is the most common genetic cause of intellectual disability, with a prevalence of 1 in 700 live births in the USA (Mai et al., 2019). DS is characterized by phenotypes that impact many organ systems throughout the body and can result in shortened stature, craniofacial abnormalities, delayed motor skill acquisition, neuroanatomical changes during both prenatal and postnatal development, and cognitive impairments (Antonarakis et al., 2020). Although many studies have identified and described the various phenotypes associated with DS, the basic underlying molecular mechanisms that contribute to many of these differences are still under investigation. The goal of this growing compendium of foundational knowledge is to enable identification of therapeutics and treatments, especially for the cognitive changes.

To better understand the changes occurring in DS, the field has long turned to mouse models as an experimental system. In particular, the Ts(17^16^)65Dn (Ts65Dn) mouse, developed in 1990, was the first trisomic mouse model of DS that survived to adulthood enabling postnatal studies (Davisson et al., 1990). Ts65Dn is the result of a translocation of the distal region of mouse chromosome 16 (MMU16), which is syntenic to HSA21, onto the centromeric region of MMU17. This freely segregating marker chromosome carries ∼119 genes triplicated from mouse chromosome 16, as well as ∼60 genes from MMU17 that are not triplicated in people with DS (Duchon et al., 2011; Reinholdt et al., 2011). The resulting Ts65Dn model partially replicates both the aneuploidy and triplication of genetic material that is seen in the majority of DS cases (Hsu 1998; Shin et al., 2010).

The Ts65Dn mouse exhibits many of the phenotypes present in people with DS (Davisson et al., 1993; Davisson et al., 1990), including delayed acquisition of motor skills and gross motor dysfunction, as well as cognitive impairment (Vicari, 2006; Olmos-Serrano et al., 2016a,b; Davis and Kelso, 1982; Latash and Corcos, 1991; Shumway-Cook and Woollacott, 1985; Aziz et al., 2018, 2019; Costa et al., 1999). Intellectual disability is the most penetrant feature of DS and can manifest in a number of ways, including deficits in spatial learning and memory (Vicari, 2006; Chapman and Hesketh, 2000; Calesimo et al., 1997; Nadel, 2003). These changes in cognition are due to structural and functional alterations in the brains of people with DS, including well-documented reductions in overall size and cellular density in the hippocampus and cerebellum (Olson et al., 2007; Winter et al., 2000; Guilhard-Costa et al., 2006; Guidi et al., 2008, 2011; Golden and Hyman, 1994; Kesslak et al., 1994; Aylward et al., 1997, 1999; Teipel et al., 2003). Ts65Dn mice also exhibit hypocellularity and a number of other neuroanatomical and functional differences in these areas (Lorenzi and Reeves, 2006; Insausti et al., 1998; Ayberk et al., 2004; Olson et al., 2004; Baxter et al., 2000), as well as deficits in hippocampal-dependent spatial learning and memory tasks (Olmos-Serrano et al., 2016b; Sago et al., 2000; Reeves et al., 1995; Coussons-Read and Crnic, 1996; Escorihuela et al., 1998; Martínez-Cué et al., 2002; Costa et al., 2010), suggesting that this component of DS intellectual disability can be modeled in the mice.

It is thought that changes early in neurodevelopment underlie some of the anatomical changes contributing to the intellectual disability in people with DS (Bahado-Singh et al., 1992; Contestabile et al., 2007; Guidi et al., 2008; Larsen et al., 2008; Wisniewski et al., 1984; Tarui et al., 2020) and this phenotype is replicated in Ts65Dn as well. For example, delayed neocortical expansion stemming from reduced proliferation of neural precursor cells results in a thinner developing pallial wall between embryonic (E) days 13.5 and E16.5, and an associated decrease in the medial-lateral width of the developing cortex (Chakrabarti et al., 2007). Additionally, individuals with DS have known perturbations in the development and organization of myelin (Olmos-Serrano et al., 2016a; Banik et al., 1975; Wisniewski and Schmidt-Sidor, 1989; Ábrahám et al., 2012), and similar changes have also been identified in Ts65Dn, including fewer mature oligodendrocytes and less myelin-related protein in the corpus callosum (Olmos-Serrano et al., 2016a). This close recapitulation of human DS phenotypes in Ts65Dn mice explains its wide adoption as a model for the syndrome.

Despite these similarities, a number of clinical trials that used the Ts65Dn model to gather preclinical evidence have yielded high failure rates (Gardiner, 2014; Herault et al., 2017). Therefore, the question remains as to how well Ts65Dn studies model the human condition. Of course, to be able to elucidate the cognitive and physical impacts of trisomy 21, mouse models of DS must demonstrate disease-relevant and reproducible phenotypes that are consistent over time. Although many studies over the past three decades have indicated that Ts65Dn mice exhibit DS-relevant neurological phenotypes, very rarely have studies repeated earlier seminal work to assess whether published phenotypes are still present in the current population. The originally developed 1924 line of Ts65Dn (Davisson et al., 1990) was bred on an F1 hybrid background that was a cross between C57BL/6JEi and C3H/HeSnJ mice (F1 hybrid) and contained a mutation in the *Pde6b* allele carried by the C3H/HeSnJ background. These mice were therefore prone to developing retinal degeneration, making postnatal behavioral studies difficult with the 1924 line. To address this problem, a new stock of Ts65Dn animals, the 5252 line, which contains only the wild-type allele of *Pde6b*, was developed*.* This was achieved by backcrossing C3A.BLiA-*Pde6b^+^/*J animals, which carried the wild-type allele for *Pde6b*, with C3H/HeSnJ animals for ten generations (Costa et al., 2010). This produced a congenic C3H/HeSnJ line that could then be crossed with Ts65Dn animals. In this way, the 5252 line is also maintained on a B6EiC3H F1 hybrid background to avoid changes in behavioral phenotypes due to genetic background, but do not carry the mutation resulting in blindness. However, owing to the repeated backcrossing necessary to generate the *Pde6b* wild-type C3H/HeSnJ strain, genetic variations might exist between 1924 and 5252 that contribute to phenotypic differences and drift in the lines.

**Fig. 1.**
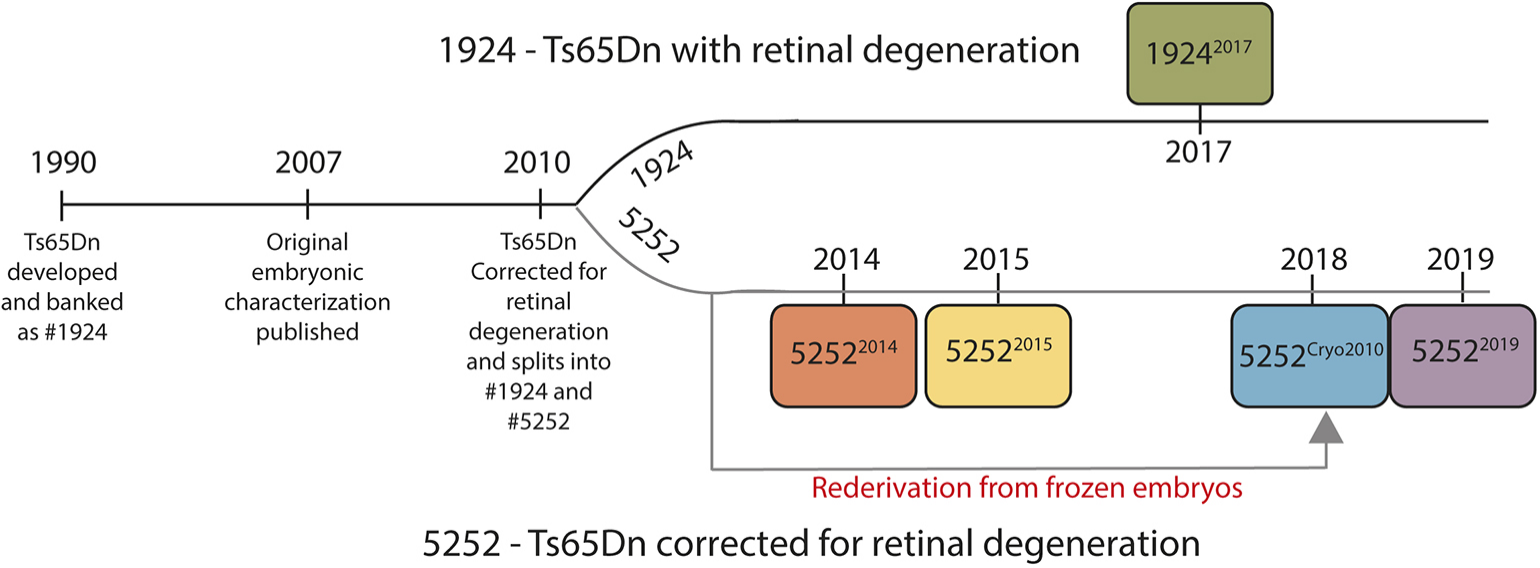
**Graphical depiction of the Ts65Dn timeline.** Ts65Dn was generated in 1990 as the first viable postnatal model of DS. In 2007, a complete embryonic characterization was published (Chakrabarti et al., 2007) describing multiple neurodevelopmental deficits. In 2010, it was reported that Ts65Dn had been corrected to contain only the wild-type *Pde6b* preventing the recessive retinal degeneration present in the original animals. Since 2010, there have been two continuously breeding strains of Ts65Dn at The Jackson Laboratory: 1924, which is the originally developed line with retinal degeneration; and 5252, a line genetically identical but without the recessive allele. The cohorts of animals analyzed in this paper are designated with their strain number and year the animals analyzed were imported from The Jackson Laboratory. The 5252^2014^ line was used to analyze the DMs and MWM reported in Olmos-Serrano et al. (2016a,b).

Our laboratory has been continuously studying the Ts65Dn mouse model for over 15 years. Throughout this time, we have occasionally had to repeat published measures and have often observed shifts in the presence and severity of phenotypes across cohorts of animals. In this report, we provide a comprehensive examination of separate cohorts of the originally generated Ts65Dn line (JAX: 1924) and the *Pde6b* corrected line (JAX: 5252) spanning over a decade to trace changes in the phenotypes (Fig. 1). In addition, we analyzed a re-derived line of Ts65Dn (5252) reconstituted from cryogenic embryo stocks that were preserved early during the development of the colony at The Jackson Laboratory (denoted here as 5252^Cryo2010^). This work is the first comparative study of multiple iterations of both strains of Ts65Dn. We measured the growth of the prenatal and postnatal brain, tested developmental milestone (DM) acquisition and spatial learning and memory using the same rigorous metrics and experimental procedures for each mouse colony. We found that Ts65Dn mice display different levels of phenotypic severity at both prenatal and postnatal stages of brain development, and that there is a large degree of variability and lability in both Ts65Dn lines across generations (Figs S1, S2). These results call into question the potential use and validity of the Ts65Dn model for studying aspects of neurological development in DS. Finally, we use these results to outline recommendations for the future use and maintenance of this model.

## RESULTS

### Embryonic gross anatomical measurements

**Fig. 2.**
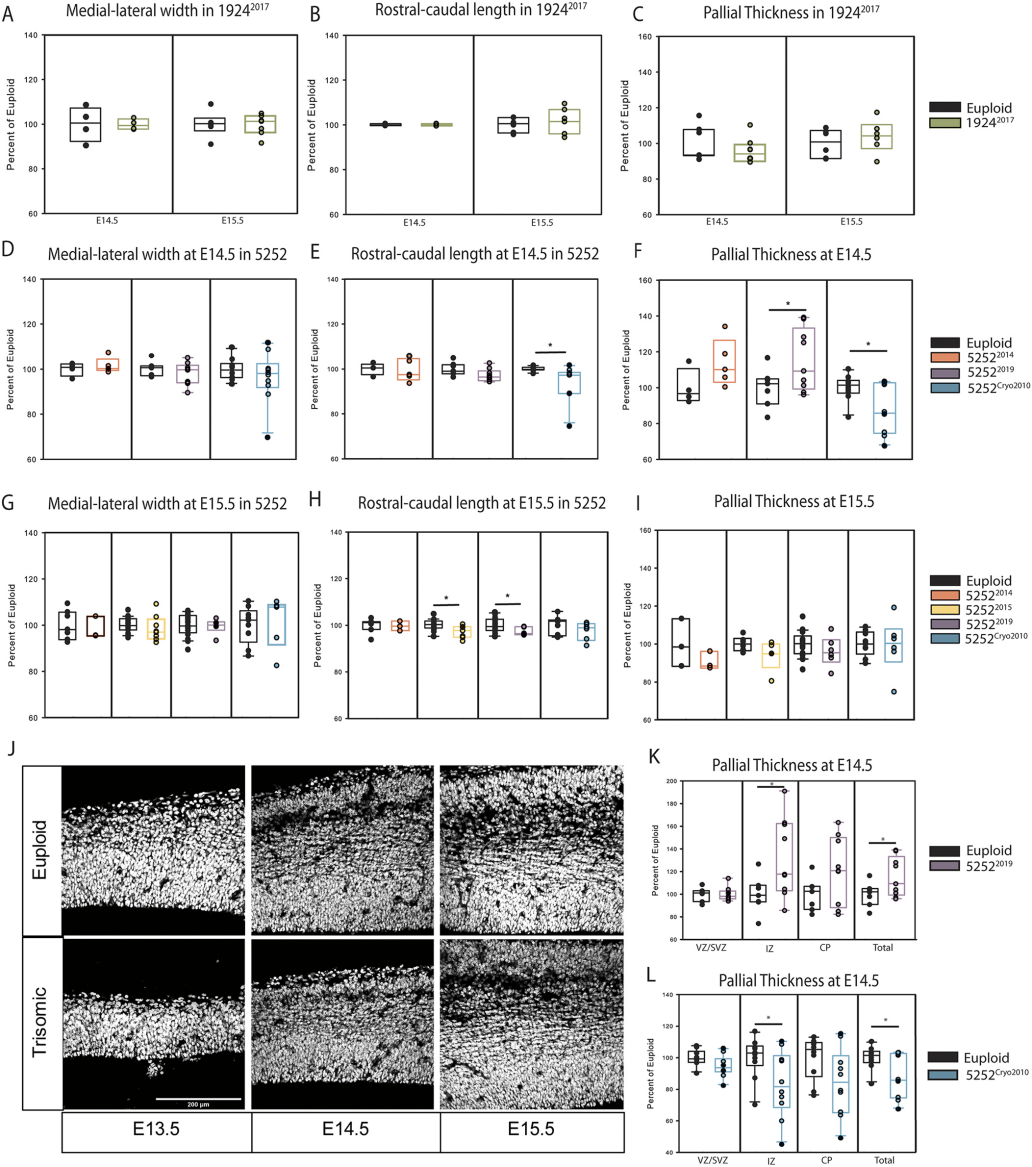
**Gross brain size and cortical thickness** **during embryonic development.** (A,B) Measurements of M-L (A) and R-C lengths (B) of the developing telencephalon in 1924^2017^ mice at E14.5 (*n*=7 and 8, respectively) and E15.5 (*n*=6 and 6). (C) 1924^2017^ pallial thickness measurements at E14.5 and E15.5. (D) M-L width measurements in the 5252 cohorts at E14.5 (5252^2014^, *n*=4 euploids and 5 trisomic; 5252^2019^, *n*=7 euploid and 9 trisomic; and 5252^Cryo2010^, *n*=10 euploid and 10 trisomic). (E) R-C length in 5252 cohorts at E14.5. (F) Pallial thickness measurements in different 5252 cohorts at E14.5. (G) M-L width in the 5252 cohorts at E15.5 (5252^2014^, *n*=3 euploid and 3 trisomic; 5252^2015^, *n*=6 euploid and 5 trisomic; 5252^2019^, *n*=21 euploid and 6 trisomic; 5252^Cryo2010^, *n*=9 euploid and 6 trisomic). (H) R-C length at E15.5 in the 5252 cohorts. (I) Pallial thickness measurements in the 5252 cohorts at E15.5. (J) Representative images of euploid and trisomic cortical walls in developing embryos at E13.5, E14.5 and E15.5 in the 5252^Cryo2010^ cohort. (K,L) At E14.5, when trisomic animals showed significant differences in total pallial thickness, both 5252^2019^ and 5252^Cryo2010^ show significant changes in the IZ but no significant differences in the VZ/SVZ or CP. All comparisons were made using an unpaired two-tailed Student's *t*-test with a probability level of *P*<0.05 (*) considered statistically significant. Box plots are mean±Q1 and Q3.

**Fig. 3.**
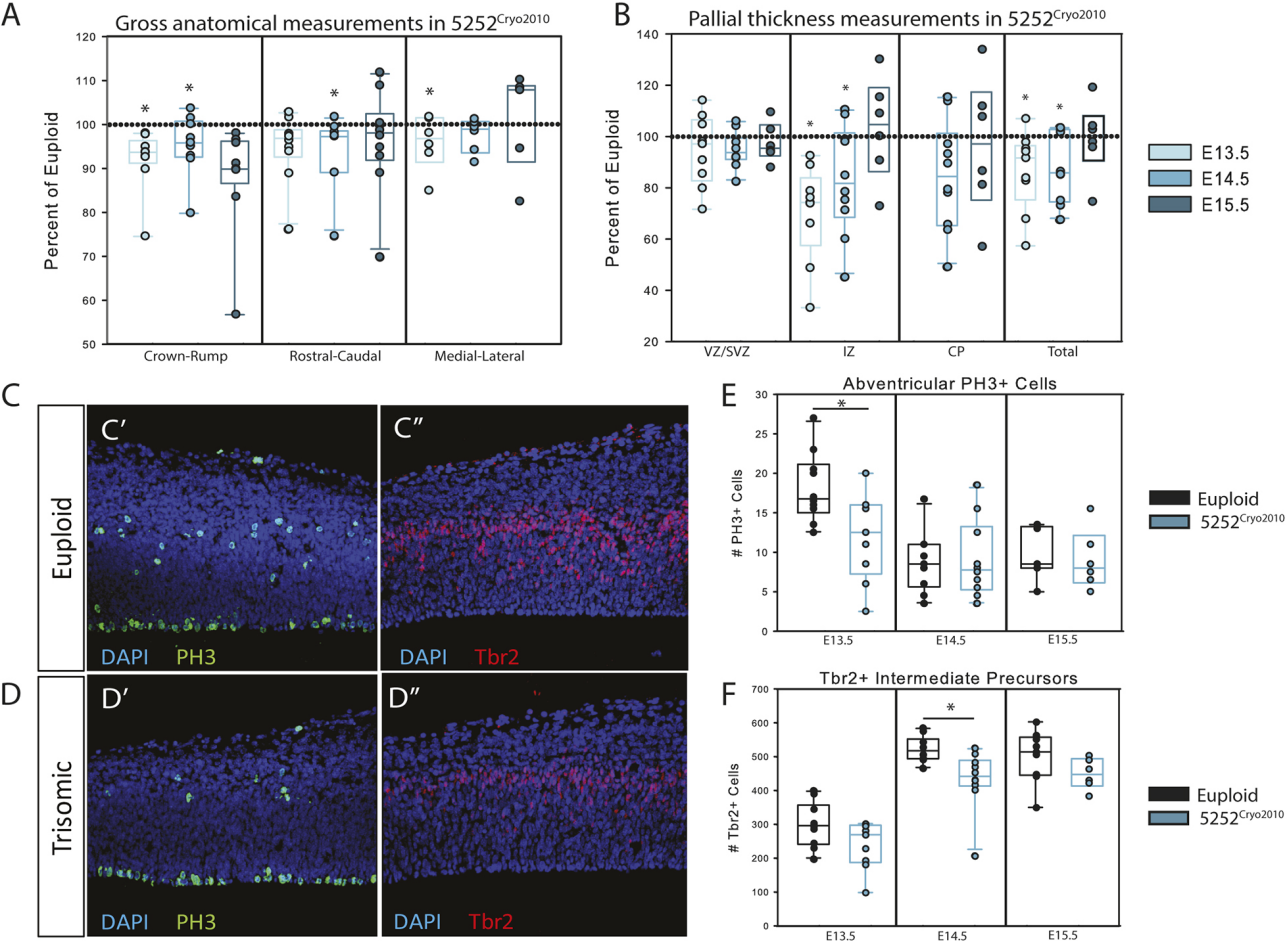
**Neurodevelopmental deficits across embryonic time in 5252^Cryo2010^.** (A) Gross measurements of 5252^Cryo2010^ mice at E13.5 (*n*=10 euploid and 9 trisomic), E14.5 (*n*=10 euploid and 10 trisomic) and E15.5 (*n*=9 euploid and 6 trisomic) compared to their euploid littermates (dashed line). (B) Thickness of the VZ/SVZ, IZ, CP and total pallium of the 5252^Cryo2010^ trisomic embryo as a percentage of their euploid littermates (dashed line) at E13.5, E14.5 and E15.5. The CP has not yet developed at E13.5. (C,D) Representative images of the developing cortex stained with PH3 to mark mitotic cells and Tbr2 to mark intermediate precursors. (E) Number of abventricular PH3^+^ cells in euploid and trisomic embryos at E13.5, E14.5 and E15.5. (F) Number of Tbr2^+^ intermediate precursor cells in euploid and trisomic embryos at E13.5, E14.5 and E15.5. All comparisons were made using an unpaired two-tailed Student's *t*-test with a probability level of *P*<0.05 (*) considered statistically significant and were conducted between trisomic embryos and their euploid controls at each time point. Box plots are median±Q1 and Q3.

The first embryonic analysis of neurodevelopmental changes in Ts65Dn was performed in 2007 using the 1924 strain (1924^2007^) and spanned embryonic ages E13.5 to E18.5 (Chakrabarti et al., 2007). This study showed that there was a significant decrease in the medial-lateral growth of the developing forebrain in the trisomic animals at E13.5, E14.5 and E16.5 but no differences in the rostral-caudal length of the cortex at any age. We repeated these gross measurements at ages E14.5 and E15.5 in each of the other Ts65Dn colonies, including 1924^2017^ [*n*=7 euploid and 8 trisomic (two litters) at E14.5; *n*=6 euploid and 6 trisomic (two litters) at E15.5], 5252^2014^ (*n*=4 euploid and 5 trisomic at E14.5; *n*=3 euploid and 3 trisomic at E15.5), 5252^2015^ (*n*=6 euploid and 5 trisomic at E15.5), 5252^2019^ [*n*=7 euploid and 9 trisomic (two litters) at E14.5; *n*=21 euploid and 6 trisomic (four litters) at E15.5], and 5252^Cryo2010^ [*n*=10 euploid and 10 trisomic (three litters) at E14.5; *n*=9 euploid and 6 trisomic (three litters) at E15.5], as well as at E13.5 in the 5252^Cryo2010^ colony [*n*=10 euploid and 9 trisomic (four litters)]. We analyzed sexes separately as well as together as initial analyses did not show any sex difference at this early developmental time point. In contrast to the original data collected from the 1924^2007^ cohort, none of the later cohorts showed a decrease in the trisomic medial-lateral (M-L) width of the forebrain at E14.5 (Fig. 2A,D) or at E15.5 (Fig. 2A,G). However, we did find a significant decrease in M-L width in 5252^Cryo2010^ at E13.5 (*P*=0.02) (Fig. 3A), which aligns with the 1924^2007^ findings.

The 1924^2007^ and 1924^2017^ animals did not show a decrease in rostral-caudal (R-C) length of the cortex at any age (Fig. 2B). However, the trisomic 5252^Cryo2010^ animals showed a significant decrease (*P*=0.045) in R-C length at E14.5 (Fig. 2E) and both the 5252^2015^ and the 5252^2019^ trisomic animals showed a significant R-C decrease at E15.5 (*P*=0.04 and *P*=0.01, respectively) (Fig. 2H). Nevertheless, after comparing all analyzed cohorts across time, there is no time point or measurement at which the trisomic embryos consistently show a deficit compared to euploid controls in gross brain dimensions.

### Embryonic pallial thickness measurements

The original differences in gross measurements in the trisomic embryos from the 1924^2007^ analysis were thought to be derived from changes in the expansion of the developing pallial wall, similar to how changes in the developing cortical wall may underlie the later microencephaly in humans with DS (Tarui et al., 2020). In particular, the 1924^2007^ cohort displayed a significantly smaller thickness of the total pallium from E13.5 to E16.5 in the trisomic embryos compared to the euploid controls (Chakrabarti et al., 2007). We therefore repeated these measurements at E14.5 and E15.5 in each cohort, as well as at E13.5 in 5252^Cryo2010^. In contrast to the 1924^2007^ results, we found no significant decrease in the pallial thickness of trisomic brains at either age in the 1924^2017^, 5252^2014^, 5252^2015^ or 5252^2019^ cohorts (Fig. 2C,F,I,K). However, similar to the original 1924^2007^ findings, the 5252^Cryo2010^ trisomic embryos did have a significantly decreased total pallial thickness at both E13.5 (*P*=0.03) and E14.5 (*P*=0.03) (Fig 2F,J, Fig. 3B) but not at E15.5 (Fig. 2I,J). Therefore, similar to the measurements of gross telencephalic growth, none of the later cohorts of Ts65Dn (e.g. 1924^2017^, 5252^2014^, 5252^2015^, 5252^2019^ and 5252^Cryo2010^) showed the exact neurohistological changes identified in the original analysis. This variability is best shown by the fact that the 5252^2019^ trisomic embryos had a significantly thicker pallium at E14.5 compared to their euploid littermates (*P*=0.048) (Fig. 2K).

### Cellular changes underlying the 5252^Cryo2010^ pallial thickness phenotype

Although the decreased pallial thickness at E13.5 and E14.5 in the 5252^Cryo2010^ animals (Fig. 3A) corresponds to the findings in the 1924^2007^ analysis, the focal changes in specific subregions of the pallium differ. Specifically, at E14.5 in the 5252^Cryo2010^ embryos, the intermediate zone (IZ) is smaller in the trisomic animals than the euploid controls (*P*=0.049) (Fig. 3B). This, along with the non-significant decreases in both the ventricular/subventricular zone (VZ/SVZ) and cortical plate (CP), drives the significant decrease in pallial thickness in the 5252^Cryo2010^ cohort. In our previous analysis of 1924^2007^ animals (Chakrabarti et al., 2007), we found significant decreases in both IZ and CP thickness that contributed to the decrease in total pallial thickness. This indicates that although the total pallial thickness might be significantly different at the same time points (E13.5 and E14.5) in the 5252^Cryo2010^ and 1924^2007^ embryos, it might be because of different underlying neuroanatomical causes.

The mechanism underlying the delayed cortical expansion in the 1924^2007^ cohort was a combination of a slower cell cycle, decreased VZ neurogenesis and slower neuronal migration (Chakrabarti et al., 2007). Partially compensating for this decreased VZ neurogenesis was an increase in the number of Tbr2^+^ intermediate progenitor cells (IPCs) from E14.5 to E16.5 (significantly increased at E16.5) that was accompanied by an increase in mitotic (pH3^+^) cells in the SVZ from E15.5 to E16.5 in the 1924^2007^ trisomic embryos (Chakrabarti et al., 2007). This increase in mitosis and neuronal output at E16.5 might explain the normalization of pallial thickness at E18.5 in the 1924^2007^ trisomic fetuses. However, in 5252^Cryo2010^, the pallial thickness of the trisomic fetuses normalizes by E15.5, despite the decreased growth at E13.5 and E14.5, again indicating that different mechanisms might be responsible for cortical expansion dynamics. Indeed, in contrast to the 1924^2007^ animals, there was a decrease in mitotic SVZ cells at E13.5 in the 5252^Cryo2010^ trisomic animals (Fig. 3C,E), corresponding to decreased numbers of Tbr2^+^ IPCs at E14.5 (Fig. 3D,F). These data indicate that decreased numbers of IPCs might be the driving force behind the decreased pallial thickness in the 5252^Cryo2010^ animals, and that it is not because of a VZ-related production shortfall as with 1924^2007^ mice. Therefore, even though both of these cohorts of Ts65Dn animals demonstrate transient cortical expansion deficits (at a gross anatomical level), somewhat recapitulating the decreased CP growth rate changes seen in humans, the mechanisms behind the phenotype are different in each Ts65Dn cohort, impeding potential translation of these mechanistic findings.

### Postnatal gross measurements and hindlimb reflex

**Fig. 4.**
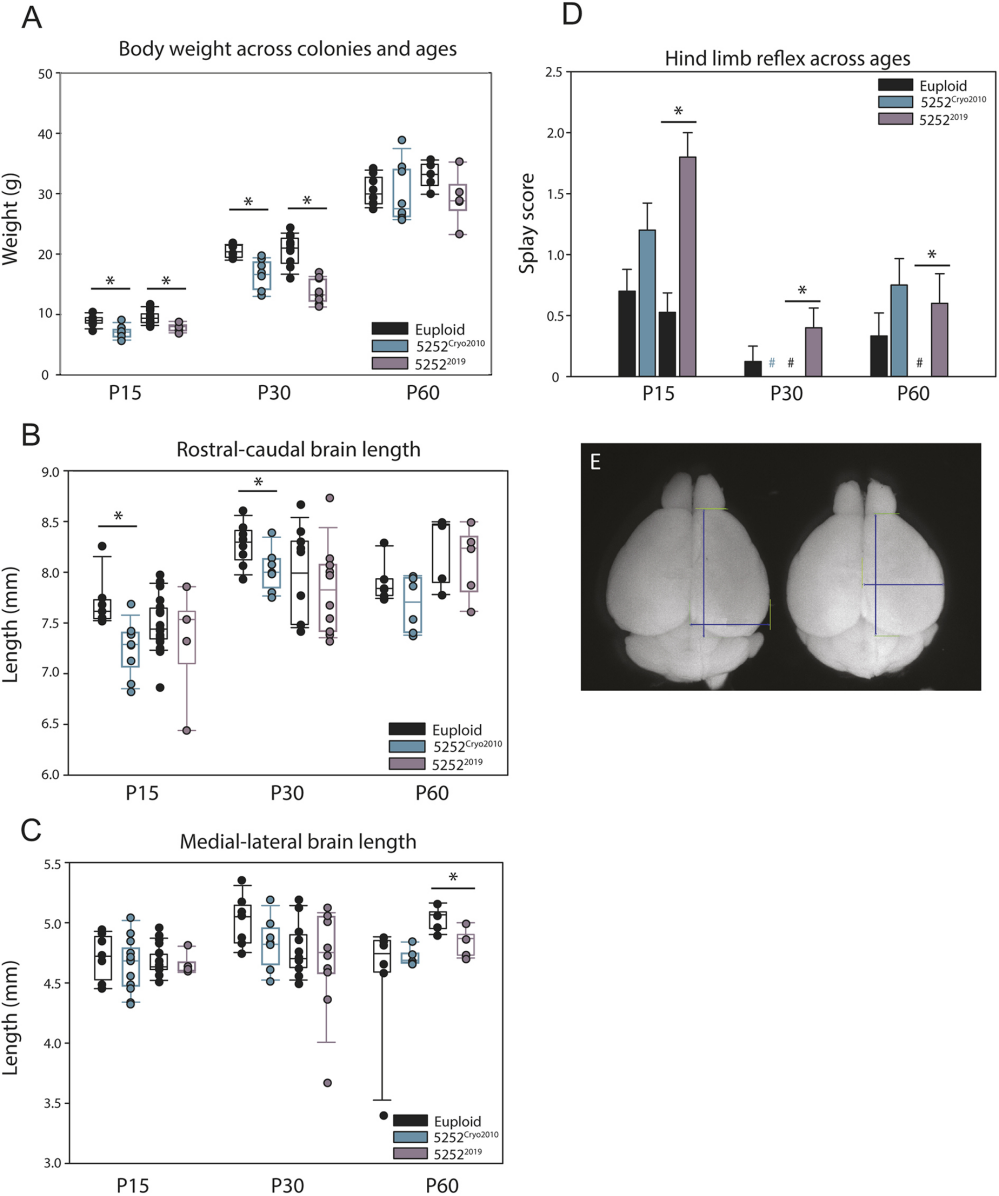
**Gross body and brain measurements across the lifespan in different colonies of Ts65Dn.** (A) Body weight of euploids and Ts65Dn at P15 (5252^Cryo2010^, *n*=7 and 9, respectively; 5252^2019^, *n*=19 and 5, respectively), P30 (5252^Cryo2010^, *n*=8 and 7, respectively; 5252^2019^, *n*=10 and 10, respectively), and P60 (5252^Cryo2010^, *n*=8 and 8, respectively; 5252^2019^ *n*=5 and 5, respectively). (B,C) R-C (B) and M-L (C) length measured as shown in Fig. 2E at P15 (5252^Cryo2010^, *n*=7 euploid and 9 trisomic; 5252^2019^ *n*=19 euploid and 5 trisomic), P30 (5252^Cryo2010^, *n*=8 and 7; 5252^2019^, *n*=10 and 10), and P60 (5252^Cryo2010^, *n*=6 and 6; 5252^2019^, *n*=5 and 5). (D) Hindlimb reflex measured at P15 (5252^Cryo2010^, *n*=20 euploid and 15; trisomic 5252^2019^, *n*=19 euploid and 5 trisomic), P30 (5252^Cryo2010^, *n*=8 euploid and 7 trisomic; 5252^2019^, *n*=10 euploid and 10 trisomic) and P60 (5252^Cryo2010^, *n*=12 euploid and 12 trisomic; 5252^2019^, *n*=5 euploid and 5 trisomic). # indicates that all animals scored a value of zero for that group. Data are mean±s.e.m. (E) Representative image of euploid (left) and 5252^Cryo2010^ (right) brains at P30, with the location of where measurements were taken indicated. Boxplots are median±Q1 and Q3, with individual data points. All comparisons were made using an unpaired two-way Student's *t*-test between trisomic and relative euploid controls, with a probability level of *P*<0.05 (*) considered statistically significant.

Individuals with DS have well-documented physical characteristics, such as smaller body size and brachycephaly. The Ts65Dn mouse model has also been shown to possess many of these gross anatomical differences (Richtsmeier et al., 2000; Hill et al., 2007; Holtzman et al., 1996; Costa et al., 2010). To determine whether these characteristics of Ts65Dn animals also vary, we assessed Ts65Dn males from the 5252^Cryo2010^ and 5252^2019^ colonies for overall body weight, and for R-C and M-L telencephalon length (Fig. 4E) at postnatal days (P) 15, 30 and 60 (Fig. 4). We found that 5252^Cryo2010^ mice were significantly smaller than controls at P15 [Fig. 4A; *n*=7 euploid and 9 trisomic (five litters); *P*=0.004] and P30 [*n*=8 euploid and 7 trisomic (four litters); *P*=0.003], but this difference normalized by P60 [*n*=8 euploid and 8 trisomic (three litters)]. Similarly, 5252^2019^ mice weighed significantly less at P15 [*n*=19 euploid and 5 trisomic (eight litters); *P*=0.005] and P30 (*n*=10 euploid and 10 trisomic; *P*<0.001) compared to euploid controls, and this also normalized by P60 (*n*=5 euploid and 5 trisomic). Interestingly, at P30 the 5252^2019^ generation was significantly smaller than the 5252^Cryo2010^ mice (reared in the same facility under the same conditions), thus showing differences between cohorts even within the same genotype (*P*=0.03). In terms of gross brain measurements, the R-C length of trisomic 5252^Cryo2010^ mice was significantly shorter compared to controls at both P15 (*P*=0.005) and P30 (*P*=0.04), but normalized at P60 (Fig. 4B); however, there was no difference in the M-L length of 5252^Cryo2010^ trisomic brains at any surveyed postnatal age (Fig. 4C). This contrasts with the 5252^2019^ animals, which exhibited no difference in rostral-caudal length at any age but did show a significantly reduced medial-lateral length at P60 (*P*=0.02).

As a measure of neurological circuit development at the behavioral level, we quantified the hindlimb reflex, which normally appears early on and is present throughout life. This behavior is scored on a three-point scale, with a score of 0 indicating a normal response of splayed hindlimbs, a score of one hindlimb indicating the retraction of one hindlimb, and a score of 2 indicating both hindlimbs are retracted when an animal is lifted by the tail. We found that 5252^2019^ trisomic males show an increase in the splay score compared to relative controls at P15 (*P*=0.001), P30 (*P*=0.02) and P60 (*P*=0.04) (Fig. 4D), suggesting an altered reflexive response in this cohort throughout adulthood. However, the hindlimb reflex of 5252^Cryo2010^ trisomic males was not alterated compared to controls at any age.

Overall, we found that postnatal gross measurements of the 5252^Cryo2010^ and 5252^2019^ Ts65Dn animals display a great deal of variability, both within cohorts as well as between Ts65Dn colonies from different founder animals. 5252^Cryo2010^ animals recapitulate many of the 1924^2007^ findings, showing a decreased body weight and smaller brain size. However, the 5252^2019^ Ts65Dn animals bred and measured years later do not exhibit any changes in brain size as measured by telencephalic length, suggesting that even within the same genotype, the severity of body and brain size changes might differ from animal to animal across cohorts.

### Oligodendrocyte maturation and myelination

**Fig. 5.**
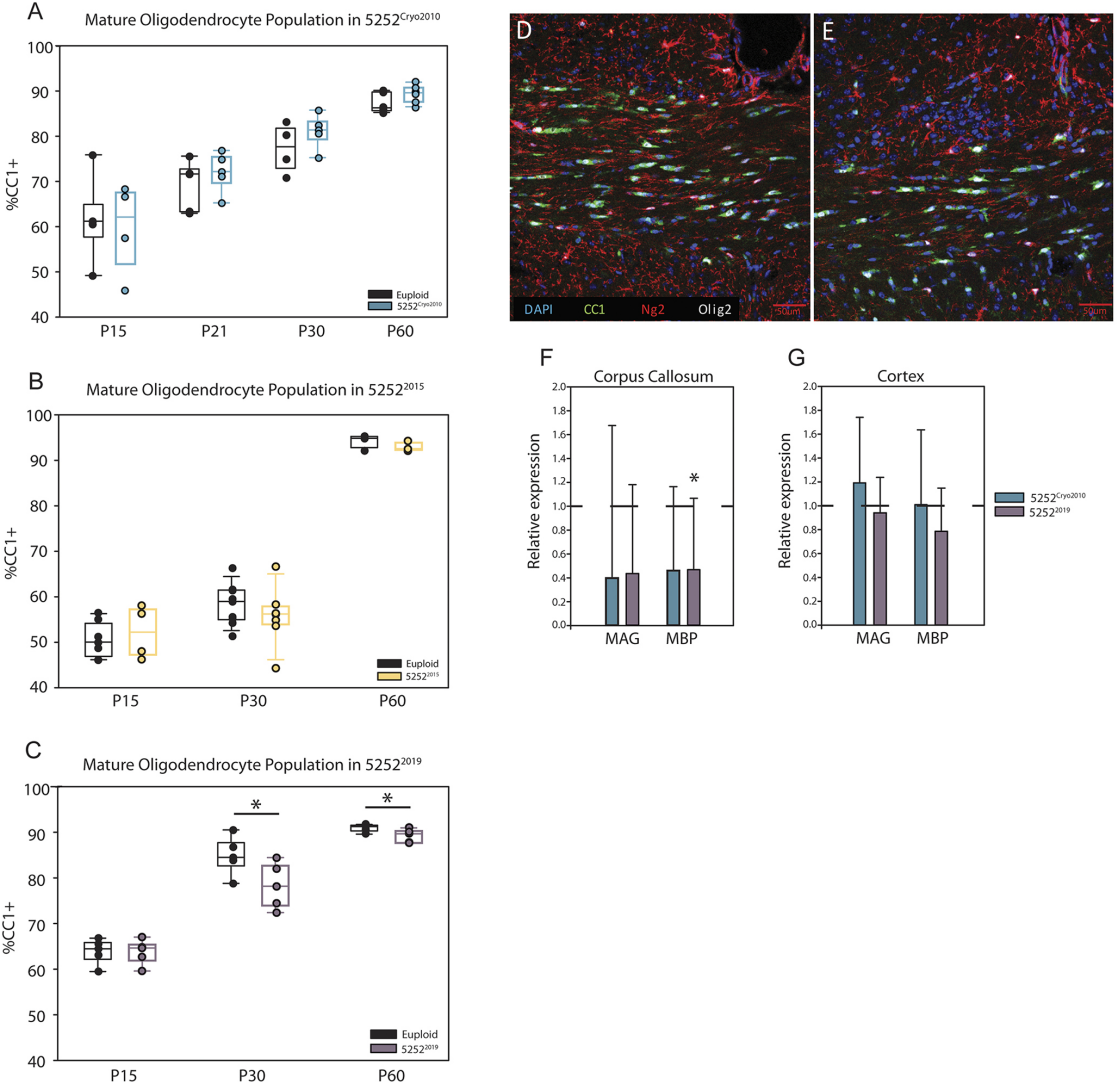
**Different iterations of the Ts65Dn colony show varied changes in oligodendrocyte maturation.** (A) Percentage of mature oligodendrocytes (CC1^+^/Olig2^+^ cells) within the corpus callosum of 5252^Cryo2010^ males at P15 (*n*=5 euploid and 4 trisomic), P21 (*n*=5 euploid and 5 trisomic), P30 (*n*=4 euploid and 5 trisomic) and P60 (*n*=6 euploid and 6 trisomic). (B) Percentage of CC1^+^/Olig2^+^ cells counted within the corpus callosum of euploid and trisomic 5252^2015^ male mice at P15 (*n*=7 and 4, respectively), P21 (*n*=9 and 7, respectively), and P60 (*n*=3 and 3, respectively), with no significant differences between genotypes. (C) Percentage of CC1^+^/Olig2^+^ mature oligodendrocytes in the corpus callosum of 5252^2019^ euploid and trisomic males at P15 (*n*=5 and 5, respectively), P30 (*n*=5 and 5, respectively) and P60 (*n*=5 and 5, respectively). (D,E) Representative images of the corpus callosum stained with CC1, Ng2 and Olig2 in both euploid (D) and 5252^Cryo2010^ (E) animals. (F,G) RT-qPCR gene expression analysis for myelin-related genes in the corpus callosum (F) and cortex (G) of 5252^Cryo2010^ (*n*=5) and 5252^2019^ (*n*=5) mice at P30, with trisomic expression levels normalized to the expression of the respective gene in euploid controls (*n*=5 and 5). Data are mean±s.e.m. Boxplots are median±Q1 and Q3, with individual data points. All comparisons were made using an unpaired two-way Student's *t*-test between trisomic and relative euploid controls, with a probability level of *P*<0.05 (*) considered statistically significant.

Previous studies have shown that the brains from people with DS have reduced levels of myelin-associated glycoprotein (MAG) and myelin-basic protein (MBP) (Olmos-Serrano et al., 2016a). Additionally, Ts65Dn oligodendrocyte precursor cells (OPCs) exhibit an intrinsic deficit in their ability to differentiate to a mature oligodendrocyte state, resulting in a decrease in myelin-related protein levels (Olmos-Serrano et al., 2016a). Specifically, this paper showed that 5252^2014^ mice had significantly fewer mature oligodendrocytes in the corpus callosum at P15 and P60, resulting in reduced protein levels of MAG and MBP in the brain. To determine the reliability of this white matter phenotype within Ts65Dn mice, we assessed the oligodendrocyte populations within the corpus callosum at multiple ages in three different generations of Ts65Dn males (Fig. 5D,E). The 5252^Cryo2010^ colony showed no change in the proportion of mature oligodendrocytes at P15 [*n*=5 euploid and 4 trisomic (five litters)], P21 [*n*=5 euploid and 5 trisomic (three litters)], P30 [*n*=4 euploid and 5 trisomic (four litters)] or P60 [*n*=6 euploid and 6 trisomic (3 litters)] compared to euploid controls (Fig. 5A). Similarly, the 5252^2015^ colony showed no changes in the percentage of mature oligodendrocytes at P15 (*n*=7 euploid and 4 trisomic), P21 (*n*=9 euploid and 7 trisomic) or P60 (*n*=3 euploid and 3 trisomic) (Fig. 5B). However, the 5252^2019^ mice (Fig. 5C) had fewer mature oligodendrocytes at P30 (*n*=5 euploid and 5 trisomic; *P*=0.05) and P60 (*n*=5 euploid and 5 trisomic; *P*=0.05), similar to the findings in 5252^2014^, illustrating how this phenotype can vary from generation to generation of Ts65Dn mice.

To confirm these temporal cohort-dependent changes in oligodendrocyte development, we measured the gene expression levels of MAG and MBP in the corpus callosum and cortex of 5252^Cryo2010^ and 5252^2019^ males with RT-qPCR (Fig. 5F,G). Expression levels of each gene (MAG and MBP) in trisomic mice were normalized to the expression level of that gene in the respective euploid control. We found no change in cerebral cortical expression levels of MAG or MBP in trisomic animals of these cohorts but we did see a significant reduction in MBP expression in the corpus callosum of 5252^2019^ trisomic mice compared to euploid controls (*n*=5 and 5, respectively; *P*=0.047). These present findings show that 5255^Cryo2010^ and 5252^2019^ trisomic mice do not recapitulate the observed human phenotype of reduced myelin-related gene expression and, taken together, these studies show how variable a major phenotype can be across generations of the Ts65Dn mice, making it difficult to compare studies across laboratories and across time.

### Hippocampal and cerebellar cell densities

**Fig. 6.**
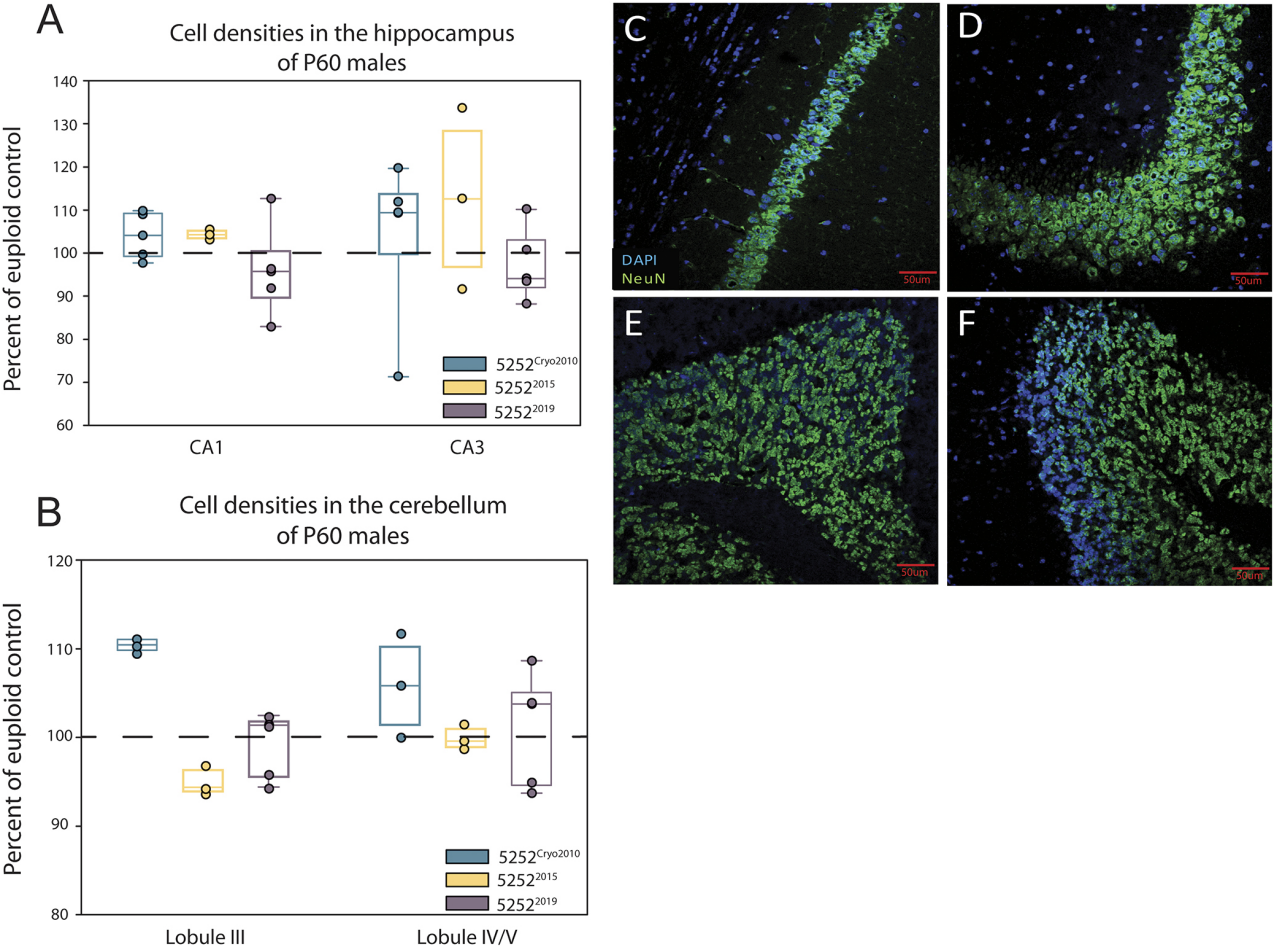
**Hippocampal and cerebellar cell densities assessed in 5252^Cryo2010^, 5252^2015,^ and 5252^2019^ males at P60.** (A) Density of NeuN^+^ cells in the CA1 and CA3 regions of the hippocampus of 5252^Cryo2010^ (*n*=5 euploid and 5 trisomic), 5252^2015^ (*n*=5 euploid and 3 trisomic) and 5252^2019^ (*n*=5 euploid and 5 trisomic) cohorts normalized to their euploid controls as a percent of control. (B) NeuN^+^ cell density in lobule III and lobule IV/V of the cerebellum of 5252^Cryo2010^ (*n*=5 euploid and 5 trisomic), 5252^2015^ (*n*=5 euploid and 3 trisomic) and 5252^2019^ (*n*=5 euploid and 5 trisomic). (C-F) Representative images of NeuN staining in CA1 (C), CA3 (D), lobule III (E) and lobule IV/V (F). Boxplots are median±Q1 and Q3, with individual data points and trisomic values normalized to respective euploid controls. All comparisons were made using an unpaired two-way Student's *t*-test between trisomic and relative euploid controls. No comparison yielded *P*<0.05.

Histological reports show that people with DS have decreased hippocampal and cerebellar volumes, and cell densities (Olson et al., 2007; Winter et al., 2000; Guilhard-Costa et al., 2006; Guidi et al., 2008, 2011; Golden and Hyman, 1994; Kesslak et al., 1994; Aylward et al., 1997, 1999; Teipel et al., 2003). It has been shown that Ts65Dn mice from the 1924 line recapitulate this phenotype in both regions, but to varying degrees dependent upon age in the hippocampus (Lorenzi and Reeves, 2006; Insausti et al., 1998; Ayberk et al., 2004; Olson et al., 2004; Baxter et al., 2000; Roper et al., 2006). To determine whether these brain regions exhibit stable reductions in size across generations of the 5252 line, we measured neuronal density in CA1 and CA3 of the hippocampus (Fig. 6A,C,D), and in lobules III and IV/V of the cerebellum (Fig. 6B,E,F) in males of three temporal cohorts of Ts65Dn mice: 5252^Cryo2010^ [*n*=5 euploid and 5 trisomic (three litters)]; 5252^2015^(*n*=5 euploid and 3 trisomic); and 5252^2019^ (*n*=5 euploid and 5 trisomic). Our results showed that none of these three studied generations of Ts65Dn exhibited a decreased cell density in CA1 or CA3 of the hippocampus nor in lobules III or IV/V compared to their euploid controls at P60. Although there were no differences between Ts65Dn mice and euploid controls within cohorts, the 5252^Cryo2010^ mice showed a significant increase in neuronal density in lobule III compared to 5252^2015^ and 5252^2019^ mice (*P*=0.01). Taken together, these data suggest that the neuronal density changes previously characterized in 1924 Ts65Dn mice are not present in all generations of 5252 Ts65Dn at P60.

### Developmental milestones

**Fig. 7.**
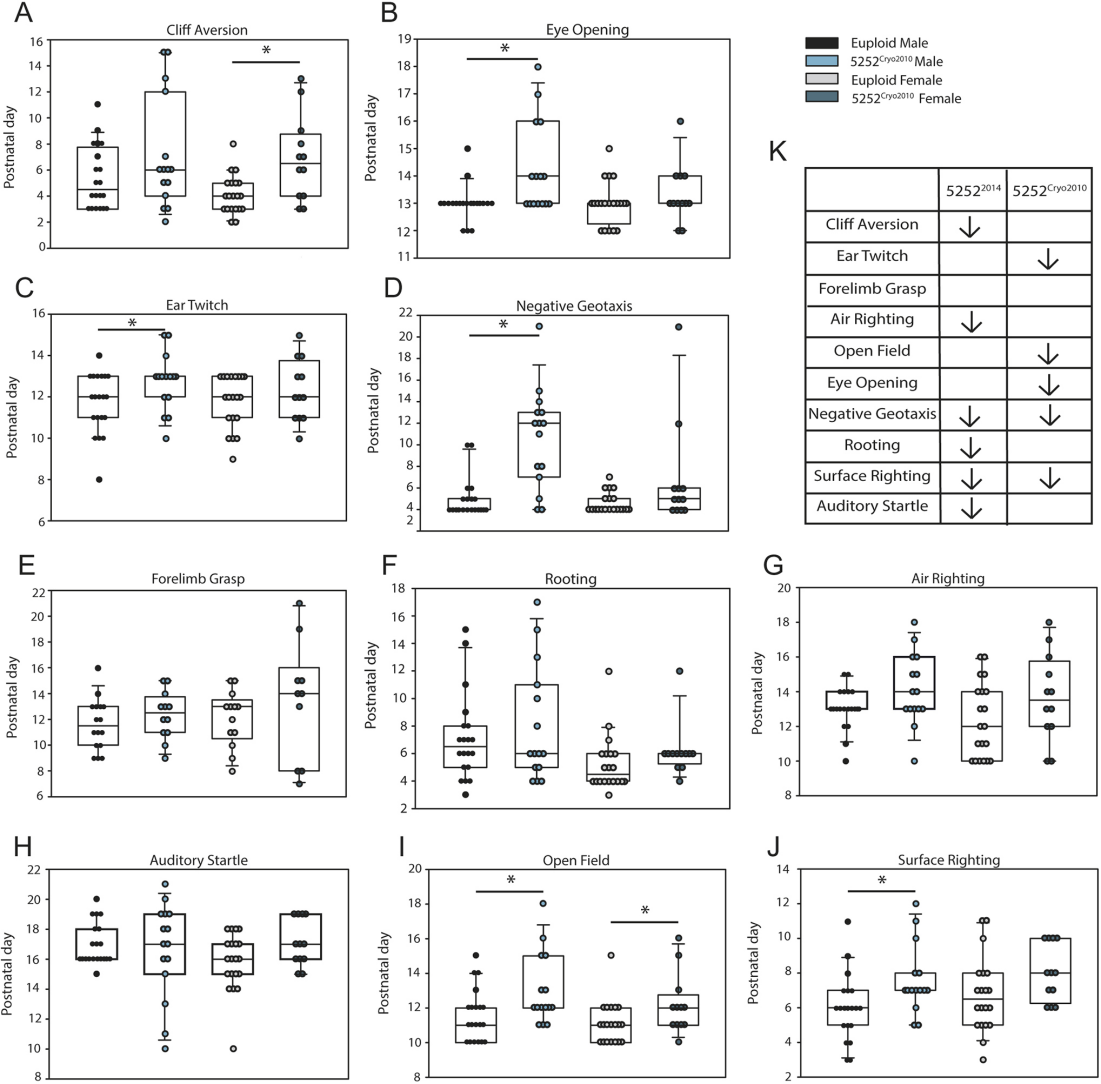
**DMs** **measured** **at** **the age animals acquired a particular skill.** (A-J) Boxplots of DM tasks for euploid males (*n*=20), trisomic males (*n*=15), euploid females (*n*=20) and trisomic females (*n*=12) from the 5252^Cryo2010^ cohort. (K) Table illustrating behaviors that were significantly delayed in 5252^2014^ Ts65Dn mice in previous publications (Olmos-Serrano et al., 2016b; Aziz et al., 2018) versus the presently studied 5252^Cryo2010^ mice. Arrows indicate behaviors that trisomic animals were significantly delayed in. Boxplots are median±Q1 and Q3, with individual data points. All comparisons were made using an unpaired two-way Student's *t*-test between trisomic animals and same-sex euploid controls with a probability level of *P*<0.05 (*) considered statistically significant.

DM tests in mice measure the acquisition of neurocognitive, gross motor and reflexive skills during early postnatal life that are akin to the acquisition of early motor and cognitive skills of human infants. Prior assessment of these skills in 5252^2014^ animals showed that Ts65Dn mice are delayed in the acquisition of surface righting, negative geotaxis, cliff aversion, rooting, air righting and the auditory startle reflex (Olmos-Serrano et al., 2016b; Aziz et al., 2018). In our study of 5252^Cryo2010^ mice, we indeed found that surface righting and negative geotaxis were delayed compared to euploid littermates (Fig. 7), but that different milestones not seen in the 5252^2014^ mice were also altered (Fig. 7A-G). Trisomic males from the 5252^Cryo2010^ colony [*n*=15 (ten litters)] were significantly delayed in the development of the ear twitch reflex (*P*=0.04) (Fig. 7C), open field navigation (*P*=0.02) (Fig. 7I), time to eye opening (*P*=0.004) (Fig. 7B), negative geotaxis (*P*<0.001) (Fig. 7D) and surface righting reflex (*P*=0.02) (Fig. 7J), compared to euploid male controls [*n*=20 (ten litters)]. However, trisomic female 5252^Cryo2010^ mice [*n*=12 (ten litters)] were only impaired in their development of cliff aversion (*P*=0.003) (Fig. 7A) and on the open field task (*P*=0.05) (Fig. 7I) compared to euploid females [*n*=20 (ten litters)], indicating a difference in phenotype severity between males and females. The results from DM testing also demonstrated the large degree of variance present within both male and female 5252^Cryo2010^ mice. Table S1 displays the calculated variance for males and females of both genotypes for all ten DM tasks. The increased variance for many tasks suggests that individual mice in Ts65Dn colonies differ from their own genotype, even within the same cohort. Importantly, these animals were tested and studied in the same laboratory and by the same methodology to avoid any confounding variables. Thus, this variability makes it difficult to draw comparisons across genotypes and across generations of a Ts65Dn strain. Additionally, this variability suggests that although the battery of tests might be useful for isolating a more general perinatal developmental delay, individual tasks cannot be used to isolate specific abnormalities.

### Morris water maze

**Fig. 8.**
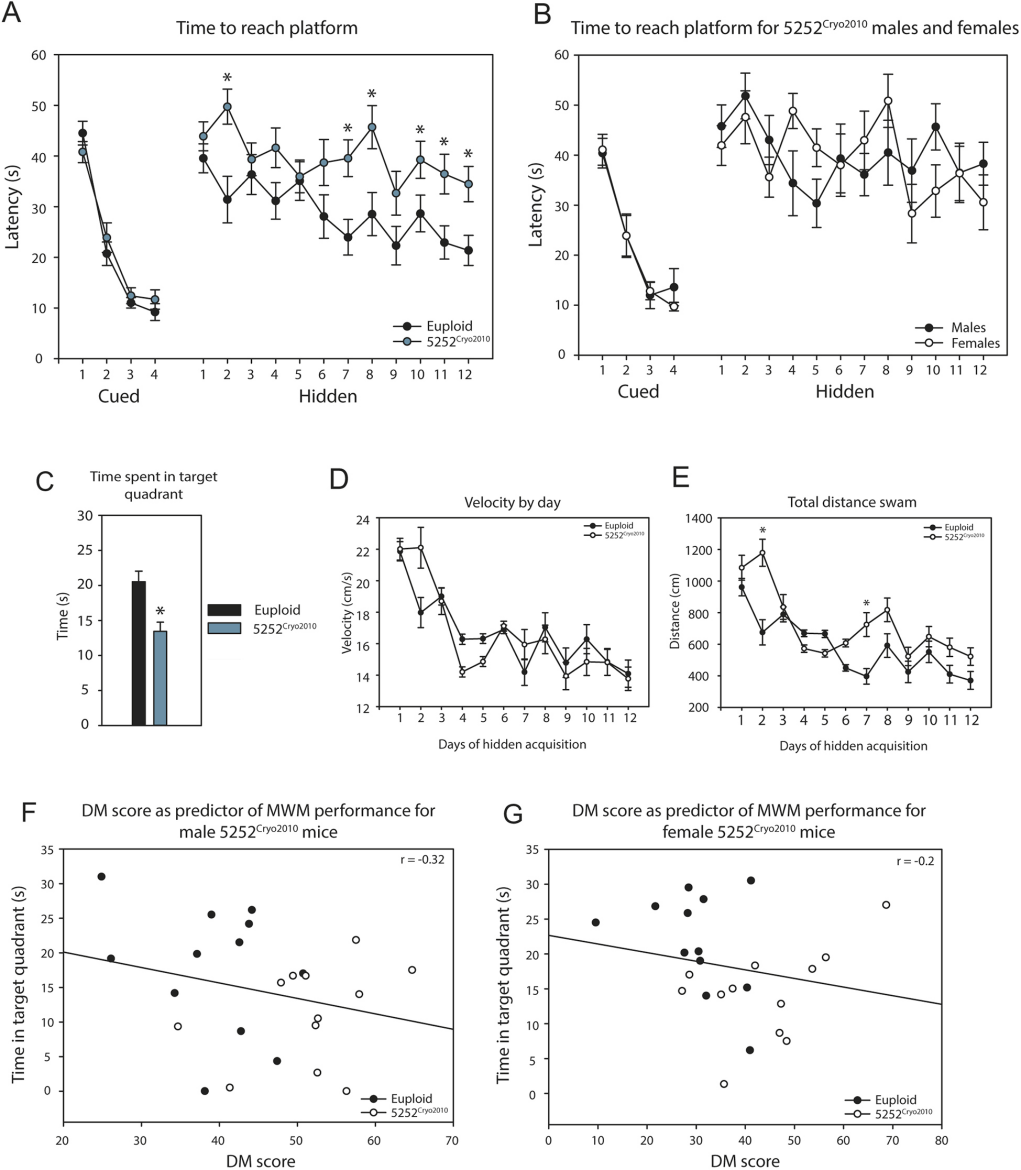
**5252^Cryo2010^ mice show impaired spatial learning on the** **MWM** **task compared to euploid controls.** (A) The latency to reach the cued or hidden platform across consecutive days of the task was significantly impacted by genotype (*n*=24 euploid and 24 trisomic, *F*_1, 46_=11.28, *P*=0.002). (B) Latency to reach platform was not influenced by sex for trisomic mice (*n*=12 euploid and 12 trisomic, *F*_1,22_=1.11, *P*>0.999). (C) Probe trial conducted after the hidden acquisition phase shows time spent in the target quadrant, with 5252^Cryo2010^ mice spending significantly less time in the target quadrant than controls (*P*=0.004). (D,E) Swimming speed and distance traveled across days during the hidden acquisition phase. There was no effect of genotype on velocity (*F*_1, 46_=0.006, *P*=0.936) but trisomic mice swam greater total distances compared to euploids (*F*_1, 46_=9.557, *P*=0.003). (F,G) Scatter plots of total time spent in target quadrant on probe trial versus cumulative DM score for males (F) and females (G). Pearson correlation coefficient calculated for each sex shows no relationship between the acquisition of DMs and performance on the MWM test. Data are mean±s.e.m. for each genotype. Repeated measured two-way ANOVAs and post-hoc Sidak multiple comparisons tests were used to compare trisomic performance to controls. A probability level of *P*<0.05 (*) was considered statistically significant for all measures.

The Morris water maze (MWM) is used to measure the ability of an animal to learn and remember spatial navigation cues, and has been a long-standing assay to study hippocampal-based learning and memory in mouse models of DS. Ts65Dn mice from the 5252^2014^ cohort learned the location of the hidden platform in an extended version of the task, but they showed an inability to relearn the platform location during the reversal phase (Olmos-Serrano et al., 2016b), suggesting that these mice exhibit altered cognitive flexibility, similar to that found in individuals with DS. To determine the consistency of this phenotype, we conducted the extended MWM task with 5252^Cryo2010^ mice to compare their performance with the 5252^2014^ Ts65Dn mice previously studied in our laboratory. In both studies, a cued learning phase was used to acclimate the mice and ensure that both genotypes were able to learn how to swim to a visible platform. Both the euploid [*n*=12 male and 12 female (ten litters)] and trisomic 5252^Cryo2010^ [*n*=12 male and 12 female (ten litters)] mice successfully learned the cued task, as indicated by a reduction in the latency to reach the visible platform over the course of the 4-day cued phase (Fig. 8A).

Following day 4 of the cued phase, both genotypes were tested for their ability to learn the location of a hidden platform using spatial cues. Both genotypes decreased the latency to reach the hidden platform over the 12 consecutive days of testing (*F*_11, 506_=4.396, *P*<0.0001, *n*=24 and 24). Genotype significantly impacted the time to reach the platform (*F*_1, 46_=11.28, *P*=0.0016, *n*=24 and 24). Trisomic 5252^Cryo2010^ mice performed similarly to euploid controls during the early learning phase of the hidden platform (days 1 to 6) but took significantly longer to reach the platform in the later phase (days 7 to 12, *P*<0.05 Sidak multiple comparisons post-hoc test), suggesting deficient learning or memory of the hidden platform location (Fig. 8A). Additionally, there were no differences between genotypes in swimming speed over the 12 days of hidden acquisition training (Fig. 8D; *F*_1, 46_=0.006, *P*=0.936, *n*=24 and 24) but trisomic mice did have greater total distances traveled compared to euploids (Fig. 8E; *F*_1, 46_=9.557, *P*=0.003, *n*=24 and 24).

To further quantify hidden platform learning, a probe trial was given after the last hidden phase training day. During the probe trial, the platform was removed, and mice swam for 60 s while time spent per quadrant was recorded. Trisomic 5252^Cryo2010^ mice spent significantly less time in the target quadrant compared to euploid controls (Fig. 8C; *P*=0.004) indicating that the 5252^Cryo2010^ Ts65Dn mice did not effectively learn the task during the hidden acquisition phase. This aligns with earlier reports from MWM studies with the 1924 line of Ts65Dn (Reeves et al., 1995) but contrasts what was previously reported for the 5252^2014^ cohort (Olmos-Serrano et al., 2016b).

The DS field has traditionally focused on studying male Ts65Dn but our results indicate that testing both sexes might provide more comprehensive insights into the effects of trisomy 21. In this study, we found no significant difference by sex in the performance of 5252^Cryo2010^ mice in the MWM (Fig. 8B; *F*_1, 22_=1.11, *P*>0.999, *n*=12 and 12) or between euploid males and females (*F*_1, 22_=0.802, *P*=0.380, *n*=12 and 12, data not shown). In line with the National Institutes of Health mandate, our data suggest that both sexes should be studied in future research to further evaluate differences between trisomic males and females.

### Developmental milestones as a predictor of adult behavioral deficits

Longitudinal studies provide insight into how trisomy can impact an animal throughout its lifespan. Our previous longitudinal study on Ts65Dn animals indicated that trisomy was a general predictor of poor brain development and function, and that DMs scores correlated with learning and memory impairments measured by the MWM test (Olmos-Serrano et al., 2016b). In this study, as the animals studied for DMs were the same as those tested in the MWM, we determined whether delays in DM acquisition were predictive of adult performance in the MWM. We calculated a composite DM score for each animal representing their overall developmental rate on all ten of the DMs. This allowed for a direct comparison across behaviors, regardless of their average age of acquisition. We found that trisomic 5252^Cryo2010^ males had significantly higher DM scores compared to euploid males (*n*=20 and 15, respectively; *P*=0.001, data not shown), indicating that they were slower to reach milestones than euploid controls. Female 5252^Cryo2010^ mice also had significantly higher DM scores compared to euploid females (*n*=20 and 12, respectively; *P*=0.004, data not shown). DM scores for both males and females were not strongly correlated with time spent in the target quadrant during the hidden probe trial (Fig. 8F,G; r_22_=−0.32, *P*=0.127; r_22_=−0.2, *P*=0.349, respectively). These results suggest that a higher cumulative DM score, representing a more delayed milestone acquisition, does not predict impaired performance in the MWM task in adulthood. This temporal connection between outcomes might be useful in evaluating the overall neurobiological impairment in a cohort of Ts65Dn animals.

## DISCUSSION

Animal models are critical tools in translational and preclinical research. However, for these models to be used for the generation of reproducible and disease-relevant findings, they must be comprehensively validated and exhibit stable phenotypes over time. The Ts65Dn model has been shown to recapitulate many of the phenotypes present in people with DS but phenotypic stability has never been addressed. Our evaluation demonstrates that the Ts65Dn mouse model of DS is variable both on a subject-to-subject basis, as well as across generational breeding cohorts. This variability makes comparisons across time and labs exceedingly difficult and has slowed progress in identifying the underlying cellular and molecular causes of DS. In this study, we highlight several well-characterized phenotypes, relevant to the formulation and execution of preclinical studies, that are subject to phenotypic variability over time. In particular, we report phenotypic drift, including the disappearance and appearance of phenotypes, in different Ts65Dn cohorts and in each Ts65Dn line (summarized in Figs S1, S2).

Phenotypic drift in the Ts65Dn colony could be due to a number of factors, making it a complicated and multifaceted concern to address. Variability within a Ts65Dn cohort could be attributable to genetic differences between animals due to their hybrid background. Additionally, these genetic differences might lead to variability between cohorts, creating ‘mini-colonies’ of animals due to selective breeding practices that use dams with higher litter success rates. These two factors might be occurring in labs breeding in-house colonies of Ts65Dn mice and account for discrepancies in published findings. Another factor contributing to phenotypic variability and drift over time is the possibility of genetic heterogeneity leading to natural evolution occurring in the Ts65Dn line as a whole. Consistent large-scale breeding of this line of animals over time could have resulted in a gradual change in Ts65Dn mice, such that the animals being studied currently are no longer the same as the version of Ts65Dn that was originally developed and characterized. Our analyses of embryonic development in 5252^Cryo2010^ mice support this conclusion. The data presented in this study provide examples of how these individual factors are contributing to the variance and phenotypic changes seen in Ts65Dn.

### Genetic heterogeneity

Early in the development of Ts65Dn, it was discovered that the line would cease to generate trisomic offspring if kept on a strict inbred C57BL/6JEiJ background (Davisson and Costa, 1999). Thus, routine breeding to maintain the colony, both at The Jackson Laboratory and in investigator labs, consists of crossing a trisomic Ts65Dn dam to an F1 hybrid male, which is the first-generation offspring from a cross between a female C57BL/6JEiJ and a male C3Sn.BLiA-Pde6b+/DnJ. As the female Ts65Dn mice are continuously backcrossed to the F1 hybrid males, the allelic combination the dams inherit and then pass on to their offspring is not consistent. In fact, each cross to generate F1 breeders results in a different admixture of alleles across the genome, and even between littermates. This genetic variance might have significant consequences on the neurological development of each animal, thus creating a wide range of phenotypic severity seen in any given cohort. For example, this allelic diversity could explain the variability seen in the DMs. In our analysis of 5252^Cryo2010^ mice, we found that trisomic mice are delayed in their overall early postnatal development (DM scores) but analysis of variance within each genotype (Fig. 7, Table S1) uncovers striking variability in the trisomic population. For eight of the ten milestones, male 5252^Cryo2010^ mice show a larger variance score compared to euploid controls, indicating that the trisomic mice have a broader range of acquisition age. This range of scores, seen specifically in the trisomic animals, could be a result of genetic differences that affect behavior and development. This same range of variance is also seen in measurements of brain anatomy at both fetal and postnatal stages (Figs 2,4). These examples illustrate that the Ts65Dn population is rather heterogeneous, even within a single cohort of animals, and that this heterogeneity impacts phenotype stability. Additionally, studies have shown that the background on which a trisomic animal is bred influences the phenotypic characterization of the animals (Deitz and Roper, 2011; Roper et al., 2020), further suggesting that the complex genetic landscape of trisomic mice is susceptible to allelic differences in backgrounds that affect phenotypes.

### Breeding strategies

Generally, labs studying Ts65Dn mice acquire breeding founders that are then used to expand and maintain their personal colonies. Only female Ts65Dn mice are fertile; therefore, the propagation of the line depends on the successful breeding of trisomic females. The long-term maintenance and in-house production of Ts65Dn mice has always been challenging as litter size tends to be small, there is prenatal loss of trisomic pups, and the trisomic dams often do not provide adequate maternal care for newborn litters (Roper et al., 2005). Thus, females that have high success rates with breeding and maternal care inevitably represent a larger proportion of any maintenance colony. With the allelic variability present in the dams, it is highly likely that genetic changes associated with increased breeding fitness might be correlated with improvement in other phenotypes manifested in offspring. This could lead to a predominance of litters that have altered or reduced phenotypic severity. Eventually, if this type of selective breeding persists over generations, it could generate ‘mini-colonies’ of Ts65Dn mice in individual laboratories. This might explain why dramatic shifts in phenotypes are observed when new breeding females are imported from The Jackson Laboratory, causing a disruption in the cycle of selective breeding. For example, when measuring the oligodendrocyte maturation phenotype, original data from Olmos-Serrano et al. (2016a,b) identified a decrease in the percentage of mature oligodendrocytes in Ts65Dn animals bred in-house between the years 2012 and 2014. However, when our laboratory measured the same phenotype with the same methodology, but from two separate sets of breeding dams (5252^Cryo2010^ and 5252^2015^), these other cohorts did not exhibit alterations in the population of mature oligodendrocytes. However, a more recent cohort (5252^2019^) did have a significantly reduced percentage of mature oligodendrocytes in the corpus callosum (Fig. 5, Fig. S2). Although the studies presented in this report may accurately represent the phenotypes of any given cohort of animals, the observed variability makes data reproducibility a challenge, even within the same laboratory, as the dams producing litters might be different. As laboratories seed Ts65Dn colonies from different founder animals and maintain their own colonies of Ts65Dn animals in-house for variable periods of time, it is likely that their animals now differ dramatically from colonies of other laboratories or from the commercial colony at The Jackson Laboratory.

These present findings uncover major difficulties for the field if the Ts65Dn line exhibits diminishment (or, as in the case of oligodendrocyte deficits, appearance, disappearance and re-emergence) of phenotypes over time. However, in some ways this variability might be more closely mirroring what is happening in people. People with DS do not have the same baseline genetics and not all have the same list or severity of phenotypes. The differences within and between cohorts of animals presented here might therefore be considered a representation of the high degree of variability and phenotype penetrance that is inherent to DS. It is possible that Ts65Dn might be accurately modeling the population diversity seen in people with DS. However, it is known that Ts65Dn mice carry ∼60 genes that are triplicated from the centromeric region of mouse chromosome 17 that are not triplicated in people with DS (Duchon et al., 2011). The effect of these non-DS-related genes on the phenotypes seen in Ts65Dn mice has rarely been addressed and could also play a role in what has been interpreted as the DS-specific phenotypic profile of this model. Because some of these MMU17 genes are known to be expressed and to play significant roles in brain development and function, it remains to be seen whether they underlie any of the Ts65Dn phenotypes described herein and in previous studies. Owing to the outlined shortcomings, we feel that the field must address these problems and establish whether there is a responsible way to use Ts65Dn and draw conclusions from these animals.

We urge the community to exercise caution and transparency when conducting research with Ts65Dn and other mouse models of DS. The ARRIVE (Animals in Research: Reporting *In Vivo* Experiments) guidelines (Kilkenny et al., 2010) provide a comprehensive set of suggestions to which all research using animal models should conform, especially with respect to publishing results. These include providing detailed reports of the study design, clear identification of experimental animals, husbandry practices and statistical methods. Based on the results in this report, we propose the following additional considerations for research using Ts65Dn mice and other trisomic models of DS. First, it is important to keep careful animal husbandry practices and documentation of parental lineage, litter size and percentage of trisomic offspring produced during the expansion of any colony. In conjunction with that, publications should state which strain of Ts65Dn is being used (1924 or 5252), the genetic background of the strains, when and from where the lines were acquired, the length of time the colony has been propagated in a given laboratory, and the number of founding breeders and F1 animals used in colony production and maintenance. Breeding schemes should have a sufficient number of trisomic dams in the colony to ensure proper diversity and avoid selective breeding. This will make comparisons of data more consistent across laboratories over time and ensure more readily reproducible findings.

Secondly, great care is required when it comes to handling trisomic animals and conducting behavioral studies. Some models of DS have an increased sensitivity to stressful or anxiety-provoking environments (Stasko and Costa, 2004). In these situations, several factors, such as the number of mice in cages, light and noise intensity in the experimental arenas, or experimenter handling, must be controlled. In many cases, all behavior for both euploids and trisomic animals should be conducted simultaneously to avoid any changing factors that could influence results and avoid pooling data from multiple sets of behavioral experiments. If this is not possible, publications should indicate the number of behavioral cohorts used to generate the sample size for a task. Additionally, reports must clearly state whether males and females were used, and how many of each were included in the study. If a sex difference is found, each sex should be analyzed separately in reference to the respective controls. In terms of data analysis, the statistical method used must be appropriate for the question being asked. Sample size or the number of litters the mice were derived from should be stated when possible. With the high degree of variance found in Ts65Dn animals, sample size can dramatically affect results and should be clearly stated in each analysis. Furthermore, the particulars related to animal housing and care may vary across facilities. The number of animals per cage and nutritional content or batch contaminants in the chow can influence rodent behavior, and should be noted when possible in publications.

Last, the field must carefully consider the questions the Ts65Dn mouse model can reliably answer and identify a common path forward if research is to continue using this model. This may include more tightly monitoring the genetic diversity of these animals and the development of a thresholding system to determine the severity of phenotypes in each animal before they enter a study. In addition to phenotype thresholding, complimentary models should be implemented when possible. This could include the use of multiple mouse models of DS to confirm a phenotype, or iPS cells to compare molecular and cellular phenotypes. It will be important to design careful genetic management procedures and standardized protocols for analysis of the newer models arriving in the DS research space, including the MAC21 and Ts66Yah mice (Kazuki et al., 2020; Birling et al., 2017). It will be critical to bear in mind that all phenotypes being studied in model systems are only relevant if also present in people with DS. When feasible, research using models of DS should be accompanied by human data to validate the findings in mice or in cells. The Ts65Dn mouse model has been a cornerstone of the DS research field, providing novel and important findings about the genetic and molecular underpinnings of trisomy 21, as well as providing a potential platform for preclinical DS research. However, our current results show that from generation to generation, within the same stock of Ts65Dn, the presence or severity of a phenotype is subject to change. Ongoing and future work using the Ts65Dn model must factor this variability into the design of experiments and the interpretation of results.

## MATERIALS AND METHODS

### Animal care and use

Different strains of the Ts65Dn mouse model of DS were purchased from The Jackson Laboratory. These strains were B6EiC3Sn.BLiA-Ts(17^16^)65Dn/DnJ (Ts65Dn; stock number 005252), B6EiC3Sn a/A-Ts(1716)65Dn/J (Ts65Dn; stock number 001924) or cryorecovered B6EiC3Sn.BLiA-Ts(17^16^)65Dn/DnJ specifically ordered from Annex 18 (Ts65Dn; stock number 005252). Ts65Dn and euploid littermates were generated by mating Ts65Dn female mice with B6EiC3Sn.BLiAF1/J (F1 hybrid; stock number 003647) males imported from The Jackson Laboratory. For routine breeding, animals were bred in harems, with females removed into individual housing as they exhibited signs of pregnancy. On average, five to ten litters were needed to generate proper sample sizes and animals being used for the same sets of experiments were produced at the same time from multiple females. Multiple different cohorts of Ts65Dn mice were imported from The Jackson Laboratory from 2014 to 2019. For clarity, the different colonies are named based on the Ts65Dn strain and time of importation (Fig. 1): 1924^2017^ (imported from Jackson Laboratories in 2017 and bred for less than five generations; strain 001924), 5252^2014^ (imported from Jackson Laboratories in 2014 and bred for less than ten generations; strain 005252), 5252^2015^ (Imported from Jackson Laboratories in 2015 and bred for five to ten generations; strain 005252), 5252^2019^ (imported from Jackson Laboratories in 2019 and bred for less than five generations; strain 005252) and 5252^Cryo2010^ (Strain 5252 recovered from cryopreserved embryos frozen around 2010, imported in 2019, and bred for five to ten generations). Genotyping to confirm trisomy was performed using genomic DNA from tail snips, using primers that have been previously described (Reinholdt et al., 2011): trisomic primers, Chr17fwd-5′-GTGGCAAGAGACTCAAATTCAAC-3′ and Chr16rev-5′-TGGCTTATTATTATCAGGGCATTT-3′ (these primers produce a 275 bp amplification product); and positive control primers, IMR8545-5′-AAAGTCGCTCTGAGTTGTTAT-3′ and IMG8546-5′-GGAGCGGGAGAAATGGATATG-3′ (these primers produce a 600 bp product from the Rosa locus). PCR cycle conditions were: step 1: 95°C for 2 min; step 2: 95°C for 20 s; step 3: 55°C for 30 s; step 4: 72°C for 45 s (steps 2-4 repeated for 40 cycles); step 5: 72°C for 5 min, followed by a 5 min extension at 72°C and a 4°C hold. PCR products were separated on a 1% agarose gel.

All murine experiments were conducted according to international ethical standards and approved by the Institutional Animal Care and Use Committees of Boston University and the National Institutes of Health guide for the care and use of laboratory animals. All experimental animals were bred and housed in a pathogen free facility with weekly wellness assessments conducted by veterinary staff. Animals were housed in individually ventilated cages with standard bedding and a nestlet square. Rodent chow and water were available *ad libitum*. The colony was maintained on a 12:12 light/dark cycle, with lights on at 07:00 h. For the DMs, animals were housed with littermates and mother until weaning, when they were then housed in cages of three to five animals, regardless of genotype. All animals were separated into single cages 2 weeks before MWM testing, and remained that way for the duration of the study.

### Tissue collection

Breeding pairs were established so that vaginal plugs could be checked twice daily. The presence of a vaginal plug was designated as E0.5. A 10% weight gain at E10 was used to confirm pregnancy (Johnson et al., 2010). Prenatal studies were performed at E13.5, 14.5 and E15.5, whereas postnatal animals were collected at P15, P21, P30 and P60.

Both male and female embryos were extracted for analysis and were fixed for 24 h in 4% paraformaldehyde (PFA) at 4°C. Fixed tissue was then washed three times in 1× PBS and used for gross anatomical measurements. For postnatal tissue collection, animals were anesthetized by intraperitoneal injection of a ketamine/xylazine cocktail and intracardially perfused with 4% PFA in 0.1 M PBS (pH 7.4). Perfused brains were removed and postfixed in 4% PFA overnight at 4°C. Postfixing, tissue was placed in 30% sucrose for 24 h at 4°C, and embedded in Tissue-Tek optimal cutting temperature compound (OCT; Sakura). Embedded tissue was frozen rapidly on dry ice and 100% ethanol, and either stored at −80°C or immediately sectioned (16 µm) using a Microm HM 550 (Thermo Fisher Scientific). The sections were mounted on Superfrost Plus slides (Thermo Fisher Scientific). Slides were dried at room temperature then stored at −80°C.

### Gross anatomical measurements

Embryos and postnatal brains were imaged using an Olympus MVX10 microscope and measurements were made using AxioVision software (Zeiss). Embryonic crown-rump lengths were measured from the top of the head to the base of the tail. After the initial crown-rump measurements, embryonic brains were dissected out from the cranium and imaged as well. Cortical rostral-caudal lengths were measured from the frontal pole to the caudal aspect of the developing telencephalon, and cortical medial-lateral lengths were measured from the median longitudinal fissure to the maximal lateral aspect of the telencephalic hemisphere. All embryonic measurements were normalized to euploid littermates to control for variation within each pregnancy before genotype averages were calculated and compared using an unpaired two-tailed Student's *t*-test.

### Neocortical layer measurements

In order to visualize the neocortical layers, the embryonic brain sections were stained with TO-PRO-3 following the manufacturer's instructions (Invitrogen) and mounted in Vectashield with DAPI (Vector Laboratories) before being imaged via confocal microscopy. For each sample, two *z*-stack images at the level of the future somatosensory cortex in each hemisphere were acquired with a Zeiss LSM 710 microscope system using Zen software. Using LSM Image Browser software (Zeiss), the thickness of the VZ and SVZ, the IZ, the CP, and total pallial thickness for each image, was measured. The measurements were averaged for each sample and normalized to the mean of euploid littermates. Measurements were then averaged by genotype and compared using an unpaired two-tailed Student's *t*-test.

### Immunohistochemistry and quantification

Slides containing the cut tissue were brought to room temperature and rehydrated in 1× PBS for 15 min. Slides were then washed three times in 1× PBS for 5 min each and incubated in a blocking solution (5% normal goat serum, 0.3% Triton X-100 and 1× PBS) for 1 h at room temperature. This was followed by incubation in primary antibody overnight at room temperature. Slides were washed three times in 1× PBS and incubated with fluorescent appropriate secondary antibodies in blocking solution for 1 h at room temperature. Finally, slides were mounted in Vectashield with DAPI. The following primary antibodies were used: rabbit anti-oligodendrocyte transcription factor 2 (embryonic, 1:300; postnatal, 1:250; Millipore, AB9610), rabbit anti-cleaved caspase 3 (1:500; Cell Signaling Technology, 9661-s), mouse anti-phospho-histone 3 (1:500; Millipore, 06-570), rabbit anti-Tbr2 (1:500; Abcam, 23345), guinea-pig anti-NG2 (1:1000; gift from William Stallcup, Sanford Burnham Prebys Medical Discovery Institute, San Diego CA, USA, RRID: AB_2314937), mouse anti-CC1 (1:500; CalbioChem, OP80) and mouse anti-NeuN (1:500; Millipore, MAB377). Secondary antibodies were AlexaFluor 488-, 546- and 633-conjugated (embryonic, 1:500; postnatal, 1:250, Invitrogen).

All sections were mounted with Vectashield with DAPI and imaged using a LSM 710 confocal microscope (Zeiss). For OLIG2^+^, CC1^+^ and NG2^+^ cell quantification, three to four images of the corpus callosum from each animal were taken at 20× magnification and 16 μm confocal *z*-stacks were analyzed using LSM software. Cell densities in the hippocampus and cerebellum were assessed by taking an average of five 16 μm *z*-stack confocal images at 20× magnification of the CA1 and CA3 of the hippocampus and lobule III, and IV/V of the cerebellum. The total number of NeuN^+^ cells were counted and normalized to the volume of the image.

### Developmental milestone and hindlimb reflex

Male and female pups were tested on a set of neonatal behavioral assessments to measure developing sensory and motor skills that included: (1) body orienting and motor coordination (surface righting, air righting and negative geotaxis); (2) strength (cliff aversion and forelimb grasp); (3) sensory system development (rooting, auditory startle, ear twitch and eye opening); and (4) rotatory behavior in open field. All experimental procedures were performed between 10:00 h and 14:00 h daily by the same experimenter, in a blind set up to avoid experimenter bias of genotype. At the start of the DM procedures, all pups were removed from parent housing to a clean cage. Pups were assessed one at a time and returned to the parental cage after task completion. Animals were tested daily between P0 and P21, with weight data collected at P0 and forelimb/hindlimb tattoos made using a sterile needle filled with non-toxic green ink to identify individual pups throughout the course of the study. The data collected were in the form of the amount of time (latency) to exhibit the presence or absence of the given reflex. If a pup exhibited the reflex for two consecutive days, the milestone was scored as acquired and no longer tested in that pup on subsequent days. A DM score was calculated for each animal using the following formula: 1+(X-A)×(10-1)/B-A, where X is the individual score, A is the lowest score and B is the highest score for a given milestone. Males and females were evaluated separately; therefore, score ranges were based on each respective sex. These were then summed for each animal to generate a composite DM score.

The hindlimb reflex was performed at P15, P21 at the end of the DMs, P30 and at P60 before the start of the MWM task. Animals were suspended by the base of the tail for a 5 s count and the degree of motor deficit in the hind legs was scored on a scale of 0 to 2. A normal extension reflex was given a score of 0, imbalanced extension or extension reflex in one limb was scored as 1, and retraction of both limbs or the absence of extension reflex in both limbs was scored as 2.

### Morris water maze

All behavioral testing was performed during the light phase between 09:00 h and 14:00 h. MWM testing was carried out to assess spatial learning. A white 125 cm diameter circular pool was filled with tap water and made opaque with the addition of non-toxic water-based white paint (Crayola). Water temperature was kept at 25°C to limit stress and hypothermia. Trials were videotaped and scored with EthoVision video tracking software (Noldus).

Male and female animals that were tested in DMs were aged to P60 for MWM behavior. The structure of the training schedule was as follows: cued trials (4 consecutive days); hidden acquisition trials (12 consecutive days); and a probe trial (1 day). During the cued and acquisition phases, mice were tested in four trials per day, with each trial beginning by placing the mouse into the water near the edge of the pool in one of the non-target quadrants. The start order was semi-random for each mouse, with a different start quadrant from the previous trial. During cued training, all visual cues were removed from sight to avoid exposure to extra-maze clues. A platform was submerged just below the water surface and was cued via a metal stick with a black ball atop, and was placed pseudorandomly in different locations across the trial days. After the completion of the cued learning phase, room cues made of cardboard with various shapes were placed on the walls surrounding the pool to facilitate spatial learning. During the hidden acquisition days, the platform remained in the same quadrant during all trials and the platform cue was removed. Mice were allotted 60 s to reach the platform. More than 60 s was scored as a failed trial, and noted as 61 s for analysis purposes. If the mouse failed to reach the platform, it was guided there by the experimenter. Once on the platform, the mouse remained there for 15 s before being removed from the pool and returned to the home cage of the subject, lined with absorbent paper towels. Parameters measured during the learning trials were latency to reach the platform, total distance swam, swimming speed, time per quadrant and time spent in the periphery of the pool.

A probe trial was tested after the completion of the hidden acquisition phase training. The probe trial was a single 60 s trial during which the platform had been removed from the pool. Mice were given 60 s to swim in the pool and time spent in each quadrant, and number of crossings into the trained platform location, were recorded.

### Gene expression analysis with qPCR

Samples for gene expression analysis with qPCR were collected by anesthetizing mice with isoflurane before decapitation. The brain was extracted and the corpus callosum and cortex were collected individually, and flash frozen. RNA was then isolated using a Qiagen RNeasy Mini Kit (74104), cDNA was prepared following the protocol for SuperScript IV VILO Master Mix (Thermo Fisher Scientific, 11756050) and quantified using a Thermo Fisher Scientific NanoDrop. RT-qPCR for MBP (Thermo Fisher Scientific, Mm01266402) and MAG (Thermo Fisher Scientific, Mm00487538) was performed following the protocol for FastTaq Advanced Assays (Thermo Fisher Scientific, 4444557). Two housekeeping genes were used for all samples, *Ppia* and *Actb.* The analysis was completed using Bio-Rad CFX Maestro software to calculate the fold change of the target genes. Trisomic expression levels of MBP and MAG were normalized to the respective gene expression in euploid controls.

### Statistical analysis

All data collection was carried out under blinded conditions in which the experimenter was blinded to the genotype of each animal until analysis. Immunohistochemical, histological and DM analyses were performed using two-way unpaired Student's *t*-tests to compare trisomic animals to their respective euploid controls, with *P*<0.05 being considered statistically significant. The boxplots presented show the median±first quartile and third quartile, with individual data points overlaid. Normal distributions for behavioral tests were determined using D'Agosino K^2^ tests. Variance was calculated for DMs using σ^2^=Σ(x-μ)^2^/N. MWM data were analyzed with repeated measures two-way ANOVAs and post-hoc Sidak multiple comparisons test.

## Supplementary Material

Supplementary information
