# Supplementary material for: Longitudinal neuroanatomical and behavioral analyses show phenotypic drift and variability in the Ts65Dn mouse model of Down syndrome
Source: Dis Model Mech. 2020 Sep 25;13(9):dmm046243. doi: 10.1242/dmm.046243 (PMC7522024; doi:10.1242/dmm.046243)
Supplement: Supplementary information [file dmm-13-046243-s1.pdf]

A

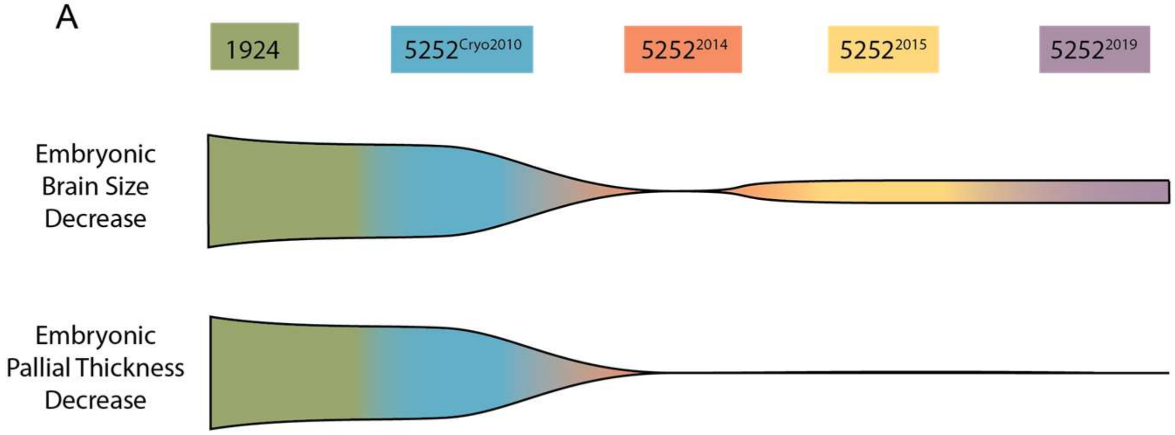

B

PRENATAL PHENOTYPES	1924					

PRENATAL PHENOTYPES	1924^{2007*}	1924^{2017}	$5252^{Cryo2010}$	5252^{2014}	5252^{2015}	5252^{2019}
M-L width	E13.5 ↓<br>E14.5 ↓<br>E15.5 –<br>E16.5 ↓<br>E18.5 –	E14.5 –<br>E15.5 –	E13.5 ↓<br>E14.5 –<br>E15.5 –	E14.5 –<br>E15.5 –	E15.5 –	E14.5 –<br>E15.5 –
R-C length		E14.5 –<br>E15.5 –	E13.5 –<br>E14.5 ↓<br>E15.5 –	E14.5 –<br>E15.5 –	E15.5 ↓	E14.5 –<br>E15.5 ↓
Pallial thickness	E13.5 ↓<br>E14.5 ↓<br>E15.5 ↓<br>E16.5 ↓<br>E18.5 –	E14.5 –<br>E15.5 –	E13.5 ↓<br>E14.5 ↓<br>E15.5 –	E14.5 –<br>E15.5 –	E15.5 –	E14.5 ↑<br>E15.5 –

Figure S1. Summary of embryonic phenotypes. **A)** Graphical representation shows the change of phenotypes measured over time with thicker width indicating a more severe phenotype present while thinner lines denoting minimal or absent phenotype. **B)** Table shows summary of findings from embryonic characterization of both 1924 and 5252 lines of Ts65Dn with ↓ indicating a decrease, ↑ indicating an increase, and – indicating no difference between trisomic and euploid controls.

*Summary of previous findings (Chakrabarti *et al.* 2007)

Disease Models & Mechanisms • Supplementary information

A

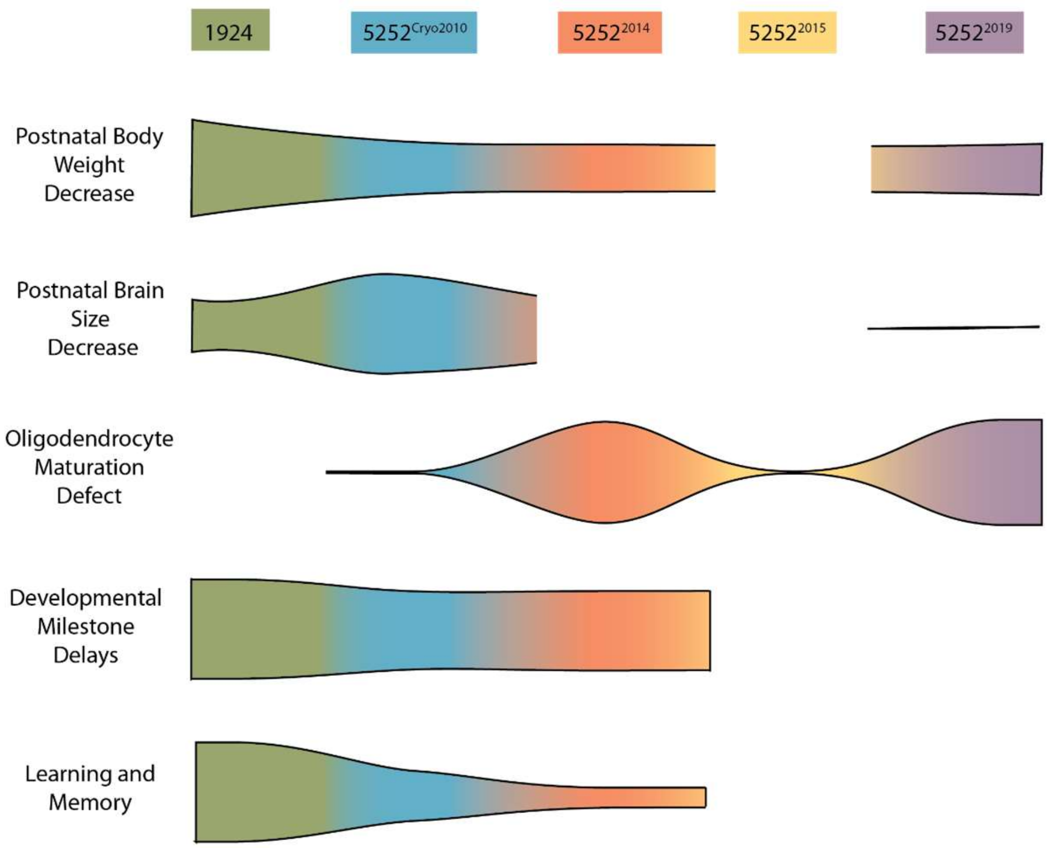

B

POSTNATAL PHENOTYPES	1924*	5252^{Cryo2010}	5252^{2014}**	5252^{2015}	5252^{2019}
Body weight	P15 ↓<br>P30 ↓<br>P60 ↓	P15 ↓<br>P30 ↓<br>P60 –	P15 ↓<br>P21 ↓		P15 ↓<br>P30 ↓<br>P60 –
Brain size	P15 – Weight<br>P45 ↓ Weight	P15 ↓ R-C<br>P30 ↓ R-C<br>P60 –			P15 –<br>P30 –<br>P60 ↓ M-L
Hindlimb reflex		P15 –<br>P30 –<br>P60 –	P90 ↓		P15 ↓<br>P30 ↓<br>P60 ↓
Hippocampal CA1/CA3 cell density	P90 –	P60 –		P60 –	P60 –
Cerebellar cell density	P35 ↓<br>P120 ↓	P60 –		P60 –	P60 –
Oligodendrocyte maturation		P15 –<br>P21 –<br>P30 –<br>P60 –	P15 ↓<br>P30 –<br>P60 ↓	P15 –<br>P21 –<br>P60 –	P15 –<br>P30 ↓<br>P60 ↓
Myelin-related gene expression		P30 –	P30 ↓ MBP/MAG (protein)		P30 ↓ MBP
Developmental milestone acquisition	8/10 ↓	6/10 ↓	6/10 ↓		
Learning and memory	↓ Hidden<br>↓ Reversal	P60 ↓ Hidden	P60 – Hidden<br>↓ Reversal		

Figure S2. Summary of postnatal phenotypes. **A)** Illustration represents the change of measured phenotypes across various cohorts of Ts65Dn with thicker line widths indicating more severe phenotypes while thinner lines represent minimal or no phenotype present. **B)** Table shows summary of findings from postnatal characterization of Ts65Dn↓ indicating a decrease or delay, ↑ indicating increase or improvement, and – indicating no difference between trisomic and euploid controls.

*Summary of previous findings in 1924 line (Reeves *et al.* 1995; Sago *et al.* 2004; Roper *et al.* 2006; Bianchi *et al.* 2010; Lorenzi & Reeves 2006; Baxter *et al.* 2000; Toso *et al.* 2008; Holtzman *et al.* 1996)

**Summary of previous findings in the 5252 line (Olmos-Serrano *et al.* 2016a,b; Aziz *et al.* 2018)

	Euploid Male		5252^{Cryo2010} Male		Euploid Female		5252^{Cryo2010} Female	
	Avg.	Var.	Avg.	Var.	Avg.	Var.	Avg.	Var.
Cliff Aversion *	5.35	5.53	7.2	17.16	4.1	2.09	6.83	9.81
Ear Twitch *	11.7	2.01	12.73	1.80	11.8	1.56	12.33	2.06
Forelimb Grasp	11.63	3.73	12.33	3.22	12.08	4.38	13.4	19.44
Air Righting	13.05	1.35	14.13	3.98	12.35	4.43	13.66	5.89
Open Field *	11.55	2.15	13.0	4.0	11.1	1.39	12.17	2.81
Eye Opening *	13.0	0.4	14.27	2.6	13.0	0.6	13.33	1.10
Negative Geotaxis *	5.0	3.2	10.6	20.11	4.6	0.84	6.83	22.64
Rooting	7.1	9.49	8.0	16.67	5.25	3.89	6.17	3.47
Auditory Startle	16.95	1.85	16.6	9.57	15.8	3.56	17.08	2.24
Surface Righting *	6.0	3.5	7.6	3.71	6.75	4.39	8.0	2.5

Table S1. Developmental milestone statistics. Table shows summary statistics for the developmental milestones from the 5252^{Cryo2010} line.
*Indicates milestone that was significantly delayed in acquisition for 5252^{Cryo2010} mice.

Disease Models & Mechanisms • Supplementary information
